# The multifaceted roles of deubiquitinating enzymes (DUBs) in pancreatic ductal adenocarcinoma

**DOI:** 10.1038/s41419-025-08052-7

**Published:** 2025-10-16

**Authors:** Min Zhou, Rongtian Wei, Jianwei Zhang, Guangbing Xiong, Jun Gong, Renyi Qin, Min Wang

**Affiliations:** 1 Department of Biliary-Pancreatic Surgery, Affiliated Tongji Hospital, Tongji Medical College, Huazhong University of Science and Technology, Wuhan, 430030 China; 2 Tongji Medical College, Huazhong University of Science and Technology, Wuhan, 430030 China; 3 Department of Pancreatic and Gastric Surgery, National Cancer Center/National Clinical Research Center for Cancer/Cancer Hospital, Chinese Academy of Medical Sciences and Peking Union Medical College, 100021 Beijing, China

**Keywords:** Deubiquitylating enzymes, Pancreatic cancer

## Abstract

Pancreatic ductal adenocarcinoma (PDAC) remains one of the most lethal gastrointestinal cancers worldwide. The diagnostic and treatment limitations have resulted in great challenges in improving the survival of patients with PDAC. Ubiquitination is an indispensable posttranslational modification involved in all cellular processes via proteolytic or nonproteolytic pathways. Deubiquitinating enzymes (DUBs) comprise ~100 proteases that remove ubiquitin from targeted proteins to regulate cellular physiological or pathological processes. Accumulating evidence has shown that DUBs are involved in the regulation of the occurrence and development of cancers. Currently, a substantial number of studies have reported that DUBs are involved in the malignant progression of PDAC. Hence, we summarize and review the latest research advances in understanding the mechanism by which DUBs are involved in modulating the development of PDAC, including proliferation, migration, invasion, metastasis, metabolic reprogramming, and chemoresistance. In addition, we present studies on how DUBs affect the immune microenvironment of PDAC and briefly characterize some therapeutic perspectives of PDAC that ultimately depend on the targeting of DUBs.

## Facts

- PDAC is one of the deadliest gastrointestinal cancers, which is characterized by an inhibitory immune microenvironment and extensive chemotherapy resistance.
- DUBs participate in almost all the biological processes of cells and especially have a pivotal role in the regulation of the occurrence and development of cancers.
- Some specific small-molecule inhibitors targeting DUBs have manifested good tumor-killing effects and acceptable safety.
- The emerging research on DUBs and their roles in remodeling PDAC immunity has revealed another potential therapeutic target.

## Open questions

- There are various DUBs manifesting modulation functions in PDAC, but which one is the hallmark of PDAC?
- Which specific lysine residues need to be further investigated, given their regulation by DUBs in PDAC?
- Can DUB be used as a prognostic marker for PDAC to make more accurate diagnoses and monitor the response to treatment in patients with PDAC?

## Introduction

Pancreatic ductal adenocarcinoma (PDAC) is a highly fatal malignancy that is projected to be the second most common cause of cancer-related mortality worldwide in the next 6 years [1, 2]. Most patients with PDAC show systemic disease at the time of diagnosis, and only ~8% of patients have a survival time of 5 years or longer after diagnosis [3]. Moreover, over three-quarters of patients suffer systemic dissemination originating from PDAC following surgery [3, 4]. Moreover, the high heterogeneity of cancer cells, pervasive desmoplasia, and complex immunosuppressive microenvironment collectively contribute to the challenging nature of PDAC treatment [5, 6]. Thus, to improve the outcome of comprehensive treatment, especially to overcome the challenges of chemoresistance, novel therapeutic strategies urgently need to be developed [7].

Ubiquitination, an essential posttranslational modification in every cellular process, regulates the destinies of proteins through either proteolytic or nonproteolytic pathways [8, 9]. As an evolutionarily conserved protein, ubiquitin (Ub) is covalently coupled to substrates to trigger a cascade of enzymatic reactions performed by the E1 (ub activating), E2 (ub conjugating), and E3 (ub ligating) enzymes during the process of ubiquitination [10]. Among these residues, the lysine (Lys) residue is the most common site that can be bound ubiquitinated by Ub [8, 11]. E1, E2, and E3 enzymes are activated sequentially, and E3 (ubiquitin ligase) enzymes, coupled with E1 and E2 enzymes, interact with the targeted proteins and catalyze the formation of a covalent isopeptide bond between the C-terminal of Ub and a substrate lysine [12–14]. Deubiquitination is the reverse regulation of the ubiquitination process, in which key peptidases termed deubiquitylating enzymes (DUBs) can remove ubiquitin chains from substrates to change their destinies [15, 16]. Currently, over 100 DUBs have been identified and are widely acknowledged to belong to 6 families based on sequence and domain conservation. These families include USPs (ubiquitin-specific proteases), OTUs (ovarian tumor proteases), UCHs (ubiquitin carboxy-terminal hydrolases), MJDs (Machado–Josephin domain-containing proteases), MINDYs (motif-interacting with ubiquitin-containing novel DUB family), and JAMMs (JAB1, MPN, MOV34 family) [17].

Disruption of the balance between ubiquitination and deubiquitination contributes to many diseases, including cancers. Hence, DUB-associated disorders result in alterations in other molecules and signaling pathways and further lead to the onset of disease. Recently, interest in the important roles of DUBs in cancer progression has increased. For example, BAP1, a member of the UCH family, has a high frequency of mutations that lead to the development of multiple malignancies, such as mesothelioma, melanoma, breast cancer, and renal carcinoma. Thus, due to the oncogenic role of BAP1 in some instances, this clinical feature is termed “BAP1 cancer syndrome” [18]. USP22, a member of the USP family, is recognized as a marker of cancer stem cells (CSCs) and was reported to promote the stemness of hepatocellular carcinoma (HCC) cells and to increase sorafenib sensitivity in mice with xenografted HCC cells [19]. Zhang et al. reported that a deficiency in the deubiquitinase OTUD1 contributes to the metastasis of breast cancer via its enzymatic activity [20]. Moreover, many researchers have focused on the indispensable roles of DUBs in PDAC. Considering recent research advancements, we summarize and discuss the mechanism of the different binding sites, variable mutations, and changeable structures of various deubiquitinases that affect the progression of PDAC.

In this review, we aim to discuss how DUBs are involved in regulating the onset and progression of PDAC and highlight the prospects for targeting DUBs to overcome the limitations of therapeutic resistance in patients with PDAC.

## DUBs as key regulators of proliferation in pancreatic ductal adenocarcinoma

**Fig. 1:**
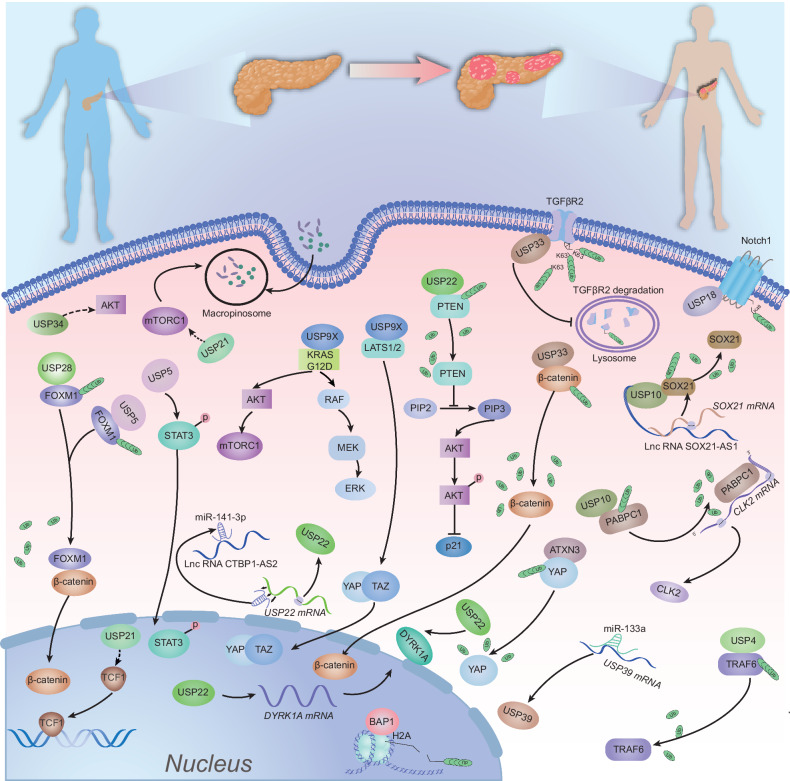
Deubiquitinases and their linked signaling cascades modulate PDAC cell proliferation. This figure highlights the regulatory role of deubiquitinating enzymes (DUBs) in PDAC proliferation by modulating oncogenic pathways. USP34 inhibits the AKT pathway, promoting tumor suppression, while USP9X activates AKT/mTORC1 and RAF/MEK/ERK pathways and enhances YAP/TAZ activity by inhibiting LATS1/2, leading to increased proliferation. USP22 regulates the PTEN/PI3K/AKT axis and DYRK1A transcription, impacting genomic stability and chemotherapy response. Additionally, USP5 and USP28 stabilize FOXM1 and regulate β-catenin, enhancing Wnt-driven proliferation. ATXN3 modulates YAP stability, and USP10 affects mRNA metabolism, while USP4 promotes inflammation via TRAF6 deubiquitination, and USP33 regulates β-arrestin-dependent GPCR signaling linked to metastasis. Overall, DUBs are crucial in regulating PDAC proliferation.

The KRAS somatic mutation that drives proliferation has been recognized as a significant hallmark of PDAC, and it is noteworthy that KRAS mutations are present in nearly 90% of PDAC cases, but the molecules involved in the interacting downstream signaling pathways have not been fully characterized. However, it is known that DUBs affect PDAC proliferation mainly through modulating the cell cycle, apoptosis, autophagy, DNA damage repair, cell stemness, and other cellular processes (Fig. 1).

Increasing evidence indicates that disorders of the Wnt/β-catenin cascade contribute to the development of some solid tumors and hematological malignancies [20]. The DUBs involved in regulating the Wnt/β-catenin pathway are associated with the proliferation of PDAC cells. Chen et al. reported that USP28 promoted cell cycle progression and inhibited the apoptosis of PDAC cells by stabilizing FOXM1, a key proliferation-associated transcription factor, to activate the Wnt/β-catenin pathway [21]. Similarly, in the Wnt pathway, USP21 interacted with and stabilized TCF7 to maintain the stemness of PDAC cells [22]. In an orthotopic pancreatic transplantation model, masses originating from USP21–HPNE cells were observed to undergo pathological changes from PanIN to PDAC. Additionally, Hu et al. discovered that USP21 promoted PDAC growth by activating mTOR signaling through binding to MAPK3 and inducing micropinocytosis to support amino acid sustainability [23]. Ubiquitin-specific protease 34 (USP34) is indispensable in various types of cancer, neurodegenerative diseases, and osteogenesis [24]. Gu et al. suggested that USP34 facilitated PANC-1 cell survival through AKT and PKC pathways [25]. Lin et al. performed an in vivo study and revealed that USP34 suppression markedly inhibited the tumor growth of PANC-1 cell xenografts in nude mice. Furthermore, USP34 was synchronously upregulated along with both PRR11 and p-p38 in PANC-1 cells [26]. By targeting FOXM1USP5, the half-life of FOXM1 was prolonged to accelerate PDAC tumor growth [27]. In a parallel study, USP5 was also shown to regulate DNA damage, cell cycle arrest, and apoptosis to promote tumor formation in PDAC [28].

Another important member of the USP subfamily is USP9X, which regulates many cellular processes [29]. USP9X dysfunction has regulated various types of malignancies by either promoting or suppressing tumorigenesis in a context-dependent manner [30]. Pal et al. established KPC (KrasLSL-G12D/+; Trp53LSL-R172H/+; Pdx1-Cre) mouse tumor-derived and human pancreatic cancer cell lines. Intriguingly, in that study, Usp9x promoted tumor cell survival and a malignant phenotype in human pancreatic tumor cells, whereas it acted as a suppressor in KPC cell-derived tumors. [31]. As a suppressor, Toloczko et al. reported that USP9X is a crucial regulator of the Hippo pathway and cooperates with LATS kinase and YAP/TAZ to impede the growth of PDAC [32]. A similar result was also verified by Zhu et al. [33]. To screen key molecules that cooperated with *Kras*^*G12D*^, Sleeping Beauty (SB) transposon-mediated insertional mutagenesis was used. PA et al. found that *Usp9x* presented the highest mutation frequency and was observed in at least 50% of PDAC tumors. USP9X was proposed to be a major tumor suppressor gene with high prognostic value and therapeutic potential in PDAC [34]. These findings collectively highlight the context-dependent dual roles of USP9X in pancreatic cancer, underscoring its critical function as both an oncogenic facilitator and a tumor suppressor, with significant implications for PDAC prognosis and targeted therapy.

USP22, the key subunit of the Spt–Ada–Gcn5 acetyltransferase complex (SAGA), is described as a marker of cancer stem cells [19]. Ren et al. discovered that USP22 was a novel PTEN-modifying enzyme. USP22 upregulated p21 expression in pancreatic cancer through PTEN–MDM2–p53 signaling, and MDM2 inhibitors enhanced the anti-pancreatic cancer effect of USP22 overexpression [35]. Bai et al. reported that USP22 was upregulated in human PDAC tissues and cell lines, where it promotes PDAC cell proliferation by increasing the level of DYRK1A—a member of the dual-specificity tyrosine phosphorylation-regulated kinases (DYRKs), which are conserved serine/threonine kinases critical to the pathogenesis of neurological disorders, particularly Down Syndrome and Alzheimer’s disease[36]. Moreover, Zhang et al.’s study found that the lncRNA CTBP1–AS2 acted as a molecular sponge by binding to miR-141-3p, which consequently increased the expression of USP22 in pancreatic cancer tissues and cell lines [37].

USP33 exerts metastatic effects on cancers such as hepatocellular carcinoma and lung cancer and contributes to the malignant phenotype of pancreatic cancer [38–40]. Liu et al. discovered that USP33 removed the K63-conjugated ubiquitin from TGFBR2, preventing its degradation, and promoted TGFBR2 recirculation to the cell membrane, amplifying TGF-β signaling. Moreover, ZEB1 in turn activated the transcription of USP33, which established a positive feedback loop to accelerate PDAC progression [39]. In addition, Zhong et al. indicated that silencing USP33 inhibited the survival and self-renewal of PDAC cells by interacting with and stabilizing CTNNB1 [41]. Ubiquitin C-terminal hydrolase L5 (UCHL5/UCH37) belongs to the family of cysteine proteases and is distributed in both the cytoplasm and nucleus [42]. Yang et al. discovered that UCHL5 promoted PDAC migration in vitro and in vivo, and supported PDAC self-renewal [43]. Ubiquitin-specific protease 4 (USP4) is located on chromosome 3 (3p21,3), and aberrant expression or activation of USP4 has been linked to various pathologies, particularly cancer [44]. By stabilizing TRAF6, USP4 activated the NF-κB signaling pathway to promote the malignant behavior of PDAC [45]. Increasing evidence has shown that USP18 is associated with the pathogenesis of cancer and that it affects the infiltration of immune cells in the tumor microenvironment. Feng et al. discovered that USP18 was significantly increased in pancreatic cancer and facilitated the progression of PDAC. Mechanistically, USP18 removed K48-linked ubiquitin chains from Notch1 to protect it from proteasome-mediated degradation, which strengthened the Notch1–c-Myc axis [46].

USP10, which has bidirectional functions, has been widely identified and recognized in several malignancies. In a study by Yu et al., USP10 was shown to be recruited by the lncRNA SOX21–AS1 to bind to and stabilize SOX21 to maintain the viability of PDAC [47]. Li et al. reported that USP10 accelerated PDAC tumor growth in vivo and in vitro by direct interaction with PABPC1 and by removal of the K27/29 ubiquitin chains linked to the RRM2 structural domain of PABPC1 [48]. In one study, Bhattacharya and colleagues found that USP10 suppression reduced pancreatic cancer cell viability, clonal growth, and invasiveness. Silencing USP10 led to increased BiP expression, which triggered endoplasmic reticulum stress and activated the unfolded protein response (UPR) and PERK IRE1α [49].

Zhao et al. discovered that elevated USP43 expression in PDAC was linked to poor overall survival and promotion of PANC-1 cell proliferation. Moreover, USP43 was involved in regulating the inhibition of CD8+ T-cell activation in PDAC [50]. USP39 serves as a critical oncogene for the viability of KRAS-dependent cancer cells. Cai et al. found that USP39 activated the AKT signaling pathway to contribute to PDAC progression. Furthermore, miR-133a directly targeted USP39 to reverse the deterioration of PDAC [51]. BAP1 mutations have been observed in various cancers, including pancreatic cancer, even at low levels of only 25% [52]. Lee et al. found that BAP1 was essential for normal pancreatic organization, and its absence, combined with the presence of KrasG12D mutation, was used to establish PDAC mouse models [53]. ATXN3, a member of the Machado–Joseph domain protease (MJD) family, has been linked to neurodegeneration and cancer. Wu et al. reported that increased ATXN3 enhanced HDAC6 expression and promoted the proliferation, migration, and invasion of PDAC cells [54]. In human breast and pancreatic cancers, Wang et al. illustrated that ATXN3 bound to WW domains within YAP1 and protected YAP1 from degradation, which then activated the downstream genes CTGF and CYR61 and facilitated tumor proliferation, migration, and angiogenesis [55].

## Modulation of PDAC invasion and metastasis by deubiquitinating enzymes

**Fig. 2:**
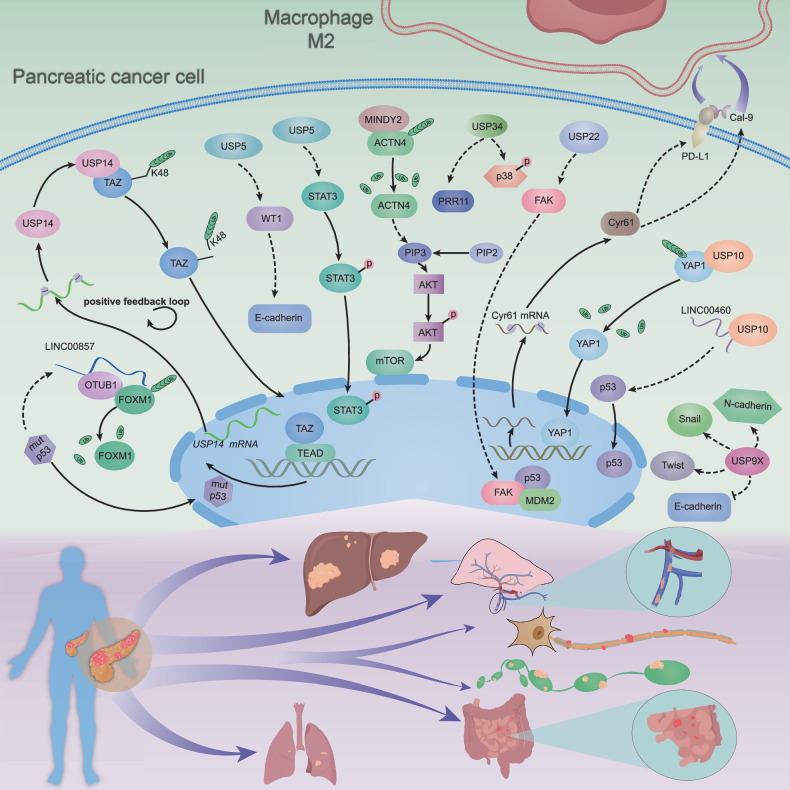
The invasion and metastasis of PDAC are governed by deubiquitinase-mediated modulation of key signaling pathways. This figure shows that deubiquitinating enzymes (DUBs) contribute to pancreatic cancer metastasis by stabilizing tumor proteins and promoting epithelial–mesenchymal transition (EMT). USP14 forms a feedback loop with TAZ/YAP1 to promote tumor growth; USP5 stabilizes FOXM1 whilst inhibiting WT1 and activating STAT3; and MINDY2 stabilizes ACTN4 and inhibits the PIP3/ OTUB1 and USP10 regulate E-cadherin/N-cadherin to promote EMT and metastasis by inhibiting PRR11 and p38 MAPK; USP34 also inhibits PRR11 and p38 MAPK to promote metastasis.

Early systemic dissemination of PDAC results in a poor prognosis and remains the principal cause of mortality in patients with PDAC [3]. Among all the metastatic niches, the liver is the most common target organ in which PDAC cell metastasis occurs [56]. The epithelial-to-mesenchymal transition (EMT) is an established key process in PDAC metastasis. During this process, DUBs play a vital role in regulating the metastatic potential of PDAC cells (Fig. 2).

According to previous studies, OTUB1 has been identified as the core member of the ovarian tumor proteases (OTU) superfamily, which tends to regulate specific ubiquitin chains, especially the K48 ubiquitin chain [57]. Intriguingly, the effects of OTUB1 in cancers are bidirectional, with both tumor-suppressive and tumor-stimulating activities [58]. In a study by Zhang et al., LINC00857 served as a scaffold to mediate the interaction between FOXM1 and OTUB1. OTUB1 deubiquitinated FOXM1 and inhibited its degradation to promote pancreatic cancer metastasis [59].

It has been widely reported that EMT is closely associated with cancer invasion and metastasis [60]. Moreover, EMT may play a more important role in the chemoresistance of PDAC [61]. Liu et al. documented that USP9X promoted the migration and invasion of PANC-1 cells through the induction of EMT and the inhibition of apoptosis [62].

USP10, a cytoplasmic ubiquitin-specific protease, stabilized p53 and contributed to pancreatic cancer development; this process is mediated by LINC00460, which acted as a scaffold [63]. In a parallel study, Liu et al. discovered that USP10 inhibited YAP1 ubiquitination, promoted Cyr61 expression, and induced immune escape, which contributed to the growth and metastasis of PDAC [64]. Xu et al. discovered that upregulated miR-103 in cancer cells promoted cell metastasis by targeting USP10 in PDAC tissues and cell lines [65]. USP22 is abnormally expressed in various cancers and plays a role in deubiquitinating PD-L1, a protein encoded by the CD274 gene in humans. Programmed cell death receptor-1 (PD-1) binds to programmed cell death-ligand 1 (PD-L1), which transmits inhibitory signals to reduce the proliferation of CD8+ T cells in lymph nodes. Additionally, PD-1 regulates the accumulation of antigen-specific T cells in lymph nodes by influencing the Bcl-2 gene, thereby affecting T-cell infiltration. [66]. In pancreatic cancer, Ning et al. found that USP22 overexpression increases ezrin, a protein that links the plasma membrane to the actin cytoskeleton. Ezrin is crucial for cell surface adhesion, migration, and organization. It has been implicated in various cancers through redistribution, phosphorylation, and cytoskeletal remodeling, which promote cell invasion and migration via epithelial–mesenchymal transition (EMT). [67].

The molecular mechanism underlying the organ selectivity of metastatic PDAC is still unknown. A previous study revealed that USP14 was closely associated with the malignant behaviors of PDAC cells, including proliferation, invasion, and migration [68]. During the progression of liver metastasis in patients with PDAC, Zhao et al. discovered that USP14 deubiquitinated and stabilized TAZ via K48-linked polyubiquitin chains. In turn, TAZ increased the mRNA level of USP14 by binding to the TEA domain transcription factor (TEAD) 1/4 response element in the promoter of USP14, which formed a positive feedback loop between USP14 and TAZ [69]. High expression of USP5 and WT1 was linked to PDAC metastasis. Degrasyn, a nonspecific inhibitor of USP5, impeded the USP5–WT1–E-cadherin signaling to restrain the onset of metastasis [70]. Furthermore, USP5 was also shown to be associated with tumor differentiation and CEA and CA19-9 levels, which were considered unfavorable factors for pancreatic cancer. In parallel, USP5 was linked to shorter overall survival, proliferation, and metastasis, which were mediated by STAT3 signaling [71].

MINDY-family DUBs are present in all eukaryotic organisms and exhibit remarkable selectivity in cleaving K48-linked polyubiquitin chains [72]. MINDY2, a member of the MINDY family, was overexpressed in pancreatic cancer tissue and was linked to a poor prognosis. Moreover, MINDY2 also had high diagnostic value and was involved in regulating the immune microenvironment. MINDY2 also stabilized ACTN4 and then activated PI3K/AKT/mTOR signaling, which facilitated the metastasis of PDAC [73]. Growing evidence has shown that CSN6, also termed COPS6, plays an important role in carcinogenesis. Amplified CSN6 is widely observed in various human cancers, including glioblastoma, colorectal cancer, breast cancer, thyroid cancer, melanoma, hepatocellular carcinoma, and pancreatic cancer [74]. In a recent study, high CSN6 expression was linked to tumor TNM stage and metastasis in PDAC patients and was shown to promote invasion and metastasis by stabilizing the c-Fos protein to induce FOXA1 expression [52].

## Metabolic reprogramming in PDAC: the emerging role of DUBs

**Fig. 3:**
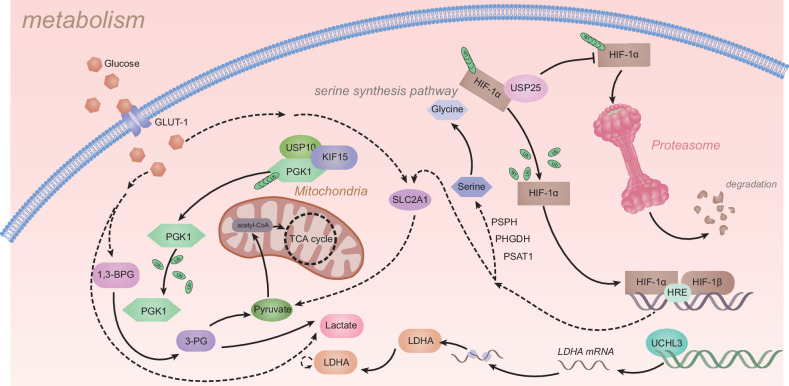
DUB-mediated signaling networks critically influence metabolic alterations in PDAC. The figure illustrates how DUBs (USP10, USP25, UCHL3) reprogram pancreatic cancer metabolism by stabilizing key glycolytic enzymes (LDHA, PGK1) and HIF-1α, enhancing glucose uptake (GLUT-1/SLC2A1) and serine/glycine synthesis (PHGDH/PSAT1). These changes suggest that DUBs are metabolic regulators during tumor progression.

Metabolism reprogramming is a pervasively self-adapting process in cancers that are relatively hypoxic and nutrient-poor. In PDAC, tumor cells adopt distinct metabolic processes, including reprogrammed glucose, amino acid, and lipid metabolism, to maintain their growth capability. Moreover, metabolic dysfunction in PDAC accounts for therapeutic resistance, including but not limited to chemoresistance, radioresistance, and immune evasion [75]. However, the underlying mechanisms of the specific molecules involved in the aberrant metabolism of PDAC are not fully understood. Many studies have manifested that DUBs play pivotal roles in the metabolic rewiring of cancers, including PDAC. KRAS mutations are pervasive in PDAC, which increases the expression of glycolysis-related genes, including GLUT1, PFK1, PGK1, and LDHA, to promote glycolytic activity and increase lactate production [76]. The DUBs USP25, USP10, and UCHL3 mainly regulate glucose metabolism to promote the progression of PDAC (Fig. 3).

Using KPCY (Pdx1-Cre; LSLKras^G12D^; Trp53^flox/flox^; Rosa26-LSL-YFP) mouse-derived organoids, PDAC patient-derived organoids, and KPCY tumor tissues, Nelson and colleagues screened specific DUBs with enzymatic activity via an activity-based proteomics technique. Furthermore, USP25 was identified as an indispensable DUB through the use of a short hairpin RNA (shRNA) system that targeted 18 DUBs to affect the growth of PDAC organoids. USP25 deubiquitinated and stabilized the HIF-1α to promote glycolysis and inhibit cell death, thus mediating metabolic reprogramming and PDAC cell survival [77]. In addition, Quan et al. first reported that KIF15 increased the stability of PGK1, a glycolytic enzyme, and facilitated deubiquitination. To clarify the process by which the deubiquitination of PGK1 is affected by KIF15, mass spectrum (MS) and co-immunoprecipitation (Co-IP) assays revealed that KIF15 recruited USP10 to deubiquitinate PGK1, which promoted glycolysis and malignant progression in PDAC [78]. Another study on glycolysis in pancreatic cancer revealed that UCHL3 promoted LDHA expression by binding to FOXM1, which facilitated PDAC cell growth [79].

In addition to glucose metabolism, amino acid and lipid metabolism also play vital roles in remodeling the microenvironment of PDAC by regulating intercellular signal transduction. The regulatory role of DUBs in amino acid and lipid metabolism in PDAC requires further investigation.

## DUB-mediated chemoresistance in PDAC: mechanisms and therapeutic implications

**Fig. 4:**
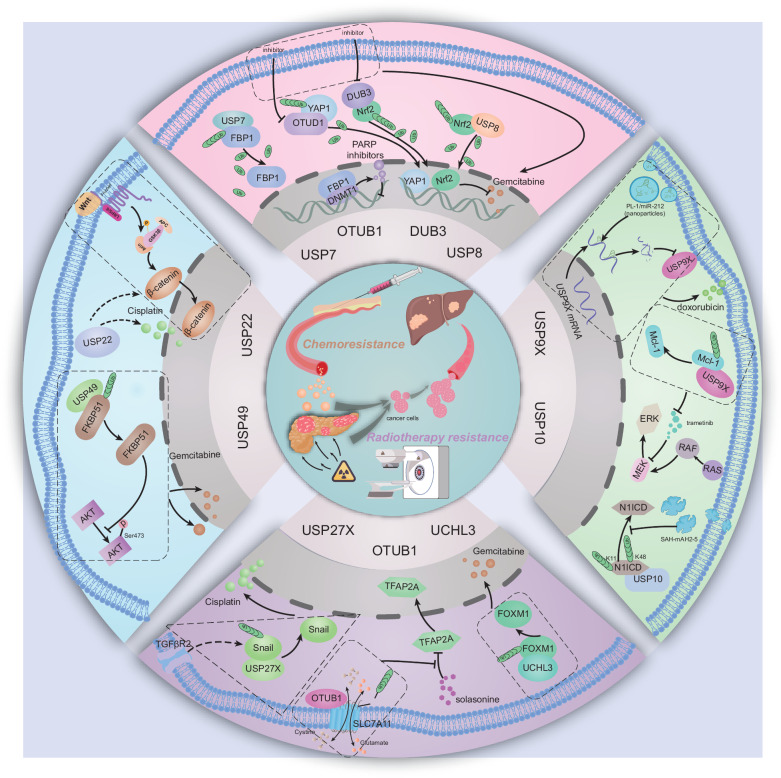
Deubiquitinases and related signaling pathways regulate the chemoresistance of PDAC. The figure demonstrates how DUBs (USP7, OTUB1, USP10, USP22, USP9X, etc.) drive chemoresistance by stabilizing DNA repair proteins (stabilizing FBP1 via USP7), activating survival pathways (inhibiting Wnt/β-catenin via USP22, activating Nrf2/YAP1 via DUB3, activating Mcl-1 via USP9X), promoting drug efflux (SLC7A11 via OTUB1) and maintaining EMT (Snail/TGFβR2 via USP10/27X) to drive chemoresistance, promote drug efflux (SLC7A11 via OTUB1), and maintain EMT (Snail/TGFβR2 via USP27X), suggesting that DUB inhibition can overcome resistance to targeted therapies.

To date, chemotherapy is widely used in patients with resectable PDAC following surgery, as well as in patients with advanced and metastatic disease [80]. The important status of chemotherapy is irreplaceable in the comprehensive treatment of PDAC. The chemotherapeutic strategies applied to patients with PDAC include gemcitabine, 5-fluorouracil (5-FU), 5-fluorouracil (5-FU)/leucovorin with irinotecan and oxaliplatin (FOLFIRINOX) and modified FOLFIRINOX (oxaliplatin, irinotecan, leucovorin, and fluorouracil) [81–83]. However, the development of pervasive chemoresistance has led to poor clinical outcomes in PDAC patients. Recently, increasing evidence has revealed the effects of DUBs on the chemoresistance of PDAC (Fig. 4).

USP7, also termed herpesvirus-associated ubiquitin-specific protease (HAUSP), has been gradually identified as a regulator of cancer hallmarks [84]. Chen et al. found that, according to mass spectrometry (MS), USP7 was associated with protein translocation in a manner that is dependent on ubiquitin-catalyzed processes. In that study, P22077, a small molecule inhibitor of USP7, suppressed cell viability and increased the chemotherapeutic sensitivity of PDAC cells to doxorubicin [85]. Furthermore, Cheng et al. revealed that the suppression of USP7 sensitized the antitumor effect of the PAPR inhibitors on pancreatic cancer. USP7 bound to and facilitated the deubiquitination of FBP1, which restrained its translocation from the cytoplasm to the nucleus. Inhibition of USP7 promoted the accumulation of FBP1 in the nucleus to facilitate the interaction between FBP1 and DNMT1, which prevented the dissociation of PARP1 from chromatin [86].

As previously described, Nrf2 and YAP are strongly associated with chemoresistance in cancers, especially via posttranslational mechanisms [87–89]. Grattarola et al. found that Nrf2 and YAP, which were associated with chemoresistance in PDAC, were relatively highly expressed in PANC-1 cells compared with the other two PDAC cell lines, MiaPaCa-2 and CFPAC-1. Silencing the Nrf2 deubiquitinase DUB3 and the YAP deubiquitinase OTUD1 inhibited cell growth and reduced the chemoresistance of PANC-1 cells to gemcitabine [90]. Cui et al. reported that USP8 bound to Nrf2 and further deubiquitinated the K48-linked polyubiquitin chains of Nrf2, which increased gemcitabine resistance in PDAC by promoting cell viability and suppressing apoptosis [91]. USP22, the key subunit of the Spt–Ada–Gcn5 acetyltransferase complex (SAGA), is described as a marker of cancer stem cells [19]. Li et al. found that the expression of USP22 was positively correlated with the chemoresistance of PC cells to cisplatin. USP22 knockdown reversed this effect by suppressing the Wnt/β-catenin pathway [92].

Dysfunction of USP9X has regulated various types of malignancies by either promoting or suppressing tumorigenesis in a context-dependent manner [30]. Ma et al. reported that WP1130, an inhibitor of USP9X, improved the sensitization of PC cells to gemcitabine and blocked gemcitabine-induced autophagy in PC cells [93]. Moreover, Perurena et al. first identified that trametinib, an MEK inhibitor, promoted adaptive resistance in pancreatic cancer by increasing the interaction between USP9X and Mcl-1 and that Mcl-1 stabilization was dependent on the deubiquitinating enzyme activity of USP9X [94]. In addition, Chen et al. conducted a study using nanoparticles and reported that PL-1/miR-212 decreased the expression of USP9X in PDAC cells, which thus enhanced the chemosensitivity of PDAC cells to doxorubicin [95].

Another study on the role of deubiquitinases in the resistance of PDAC cells to gemcitabine focused on USP49. Luo and colleagues discovered that FKBP51, a scaffold protein, promoted the PHLPP–AKT interaction to dephosphorylate AKT at Ser473. Through in-depth research and an understanding of FKBP51–PHLPP–AKT signaling, USP49 was found to mediate the deubiquitination and stabilization of FKBP51 to promote AKT dephosphorylation, which enhanced PC cells' response to gemcitabine [96]. To illustrate the process of ubiquitination involved in short-lived Snail-1, Lambies et al. used an siRNA screening system and reported that USP27X deubiquitinated and stabilized Snail1 to promote metastasis and decrease the sensitivity of PC cells to cisplatin [97]. Another study of aerobic glycolysis, which was involved in chemoresistance in PDAC, revealed that USP44 deubiquitinated and increased the expression of FBP1, the key enzyme involved in gluconeogenesis; this promoted the PDAC cellular response to gemcitabine by inhibiting MAPK signaling [98]. In addition to studies of classic chemoresistance in pancreatic cancer, substantial studies have focused on new applications of nontraditional antitumor medicine. For example, Liang et al. illustrated that solasonine inhibited PC cell proliferation, migration, and invasion by downregulating SLC7A11 to activate ferroptosis. OTUB1 interacted with and deubiquitinated SLC7A11, which was counteracted by the solasonine-induced dysfunction of TFAP2A, a transcription factor for OTUB1 [99]. Song et al. demonstrated that UCHL3 deubiquitinated and stabilized FOXM1 to mediate chemoresistance to gemcitabine in pancreatic cancer [100]. Recently, Zhai et al. and colleagues first discovered that the microprotein N1DARP disrupted the deubiquitination process of USP10–N1ICD, thereby promoting K11- and K48-linked polyubiquitination of N1ICD in Notch1-hyperactivated pancreatic cancer. Furthermore, SAH–mAH2–5, a cell-penetrating stapled peptide similar to N1DARP, in turn inhibited the deubiquitination of N1ICD induced by USP10. This then facilitated the degradation of N1ICD and increased the chemosensitivity of pancreatic cancer, which was verified in both organoid and KPC mouse models [101].

## Immunomodulatory functions of DUBs in the pancreatic ductal adenocarcinoma microenvironment

**Fig. 5:**
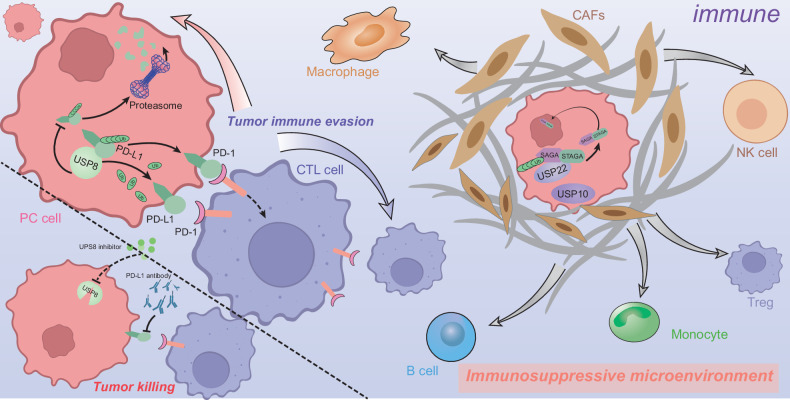
DUB-dependent signaling rewiring governs immune nich in pancreatic adenocarcinoma. This figure reveals how DUB (USP8, USP22, USP10) mediates tumor immune evasion by stabilizing PD-L1 (via USP8) and maintaining the immunosuppressive microenvironment (via USP22/SAGA/STAGA and USP10). DUB inhibition in combination with PD-L1 blockade enhances tumor killing by CTL and NK cells, thus suggesting a combinatorial immunotherapy strategy.

Pancreatic ductal adenocarcinoma (PDAC) is among the most immunosuppressed tumor types and is characterized by a prominent lack of infiltration of CD8+ T cells and lower expression of activation markers; pancreatic tumors are also termed “cold” tumors [102, 103]. Additionally, excess immune barriers are established in the process of PDAC metastasis [104]. Currently, immunotherapeutic strategies for the treatment of PDAC, including immune checkpoint inhibitors and adoptive cell transfer, such as chimeric antigen receptor T (CAR-T) cells, cancer vaccines, combinations of multiple immune agents, or other specific molecule-targeted agents, still have not achieved the expected clinical effects [105, 106]. Recently, increasing numbers of studies have demonstrated that DUBs play critical regulatory roles in cancer immunity and immunotherapy by modulating pivotal checkpoints or T-cell-associated regulators [107, 108]. Given that the critical role of DUBs is to regulate immune cell function and the immune response in the tumor microenvironment (TME), DUBs are even regarded as potential “secondary checkpoints” in cancer immunity [108] (Fig. 5).

USP8, also termed UBPY, belongs to the USP subfamily and participates in the regulation of endocytosis and protein trafficking, which is dependent mainly on its deubiquitinating activity in modulating the endosomal sorting complexes required for transport (ESCRT) [109]. USP8 was recognized as an immunomodulatory factor involved in the regulation of inflammatory bowel disease and human cancers [110, 111]. To illustrate the process of deubiquitination of PD-L1 in PDAC, Yang et al. reported that USP8 bound with PD-L1 and upregulated its expression by impairing the proteasome-mediated degradation pathway. Moreover, the combination of a USP8 inhibitor and an anti-PD-L1 agent markedly suppressed the progression of PDAC by enhancing antitumor immunity, especially via the activation of cytotoxic T cells, as verified in C57BL/6 J mouse models [112].

Another USP subfamily member, USP22, contains an N-terminal zinc finger and a C-terminal catalytic domain, while the zinc-finger ubiquitin-binding domain of USP22 does not directly link to ubiquitin because it lacks ubiquitin tail-binding pockets [113]. Many studies have confirmed the protumorigenic roles of USP22 in human cancers [113]. Li and colleagues discovered that deletion of USP22 increased the cellular response to immunotherapy and suppressed metastasis of PDAC by weakening the relative abundance of suppressive myeloid cells versus cytotoxic T cells in the pancreatic TME. USP22, along with ATXN7L3–ATXN7–ENY2 binding partners of the SAGA/STAGA complex, remodeled the immunosuppressive microenvironment of PDAC at least in part by coordinating with the PRC2 complex [114]. Through bioinformatic analyses of the TIMER and GEPIA databases, Gao et al. revealed that USP10 was associated with the infiltration of multiple immune cell types and may be involved in modulating specific immune cell subpopulations in PDAC. The USP10-related immune cell markers include CCR8 for Tregs, CD86 and CSF1R for monocytes, CD68 and IL10 for macrophages, NOS2, IRF5, and PTGS2 for M1 macrophages, and CD163, VSIG4, and MS4A4A for M2 macrophages [115].

## Limitations and perspectives

In recent years, tremendous advances have been made in the diagnosis and treatment of many cancer types. In contrast, similar success rates have not been reported in patients with PDAC, a challenging and lethal disease [1, 5]. Ubiquitination is an indispensable regulatory process that occurs in almost all signaling pathways [116]. Disorders of ubiquitination and deubiquitination lead to the occurrence of diseases, including cancers. Unsurprisingly, DUBs have been identified as the key factors associated with all the hallmarks of cancer and even as hallmarks themselves. An integrated appreciation of the physiological and pathological consequences of DUB modulation in PDAC will lead to concrete improvements in DUB-targeted therapies for patients with PDAC. Although many studies have focused on the roles of DUBs in PDAC, several challenges and concerns still need to be discussed, including the application of animal models, the mechanism of different DUB-regulated ubiquitin chains, the potential for clinical transformation and targeted therapeutic strategies.

First, the animal models used in molecular cancer research simulate the pathological processes of occurrence, development, and metastasis in cancers. However, the strength of evidence, or rather, the credibility of the research, is closely associated with the choice of animal model. Notably, experimental murine models are considered one of the most valuable tools for determining the histopathology and pharmacokinetics of PDAC [117]. Different mouse models represent various stages of PDAC progression, including tumor initiation, development, invasion, and metastasis. At least 4 sources of PDAC are available, including cell lines, organoids, mouse-derived allografts (MDAs), and patient-derived xenografts (PDXs). Following different experimental requirements, subcutaneous and orthotopic implantation can be applied in the proliferation of PDAC models. The most common and reliable models for PDAC liver metastasis are the splenic and portal vein injection models. The systemic metastasis of the PDAC model is constructed by tail vein injection. Orthotopic implantation in a PDAC liver metastasis model further simulates the changes in the tumor microenvironment of PDAC. To fully illustrate the effect of DUBs on PDAC, genetically engineered mouse models (GEMMs) could serve as sophisticated tools for the preclinical evaluation of various therapeutic approaches and could increase our understanding of disease progression. Moreover, in future studies of DUBs in PDAC, the same phenotype could be tested in at least 2 mouse models to improve the evidence level.

Second, the key features of ubiquitin are the seven lysine residues (Lys6, Lys11, Lys27, Lys29, Lys33, Lys48, and Lys63) and Met1, which can be ubiquitinated to link ubiquitination enzymes to the target substrate and can also be deubiquitinated by DUBs to detach these enzymes from the substrate [9]. K48- or K11-linked polyubiquitin chains are generally acknowledged to lead to proteasomal degradation of the target protein [118, 119]. In other words, the stabilization of the target protein by a DUB, which is dependent on its enzymatic activity, may be caused by the regulation of the K48 or K11 sites of ubiquitin. In addition, different ubiquitin chain types are closely associated with several specific signaling pathways, which may play diverse roles in the phenotype of cancer; different ubiquitin chain types are also closely associated with several specific signaling pathways, which may play diverse roles in the phenotype of cancer [120]. However, few studies of DUBs in PDAC have clarified the mechanism by which specific lysine residues are regulated by DUBs. Moreover, the modification sites of substrates regulated by DUBs have rarely been identified. The multifaceted roles of DUBs in PDAC deserve to be further explored to identify potential molecular treatment targets.

Finally, as shown in the studies above, the target proteins regulated by DUBs in PDAC mainly involve the ubiquitin-proteasome system (UPS). To date, many small-molecule inhibitors have been developed that target different components of the UPS, including DUBs, and their effectiveness is gradually being tested in cancers [121].

Unfortunately, only therapeutic targets for proteasomes, such as proteasome inhibitors (PIs), have achieved tangible success in the clinical treatment of cancer. Although several small-molecule DUB inhibitors have been identified, few have reached clinical trials in oncology. Pimozide has shown synergistic activity with cisplatin in drug-resistant NSCLC by inhibiting USP1/UAF1 complex enzyme activity [122], whereas 6MP and 6TG are specific inhibitors of PLpro and are in phase II–IV trials for the treatment of various cancers [123]. The FDA-approved drug mitoxantrone, a USP11 inhibitor that disrupts DNA repair, is one of the most promising candidates for the treatment of PDAC [124]. Undeniably, strategies targeting DUBs remain promising for overcoming drug resistance in cancer, especially in PDAC. A combination of conventional drugs and targeted DUB therapy may provide new insightful strategies for refractory PDAC, can be fully tested in multiple experimental murine models and can be used to elucidate the mechanism of the modification site.

## Conclusion

In summary, the recent studies discussed here focused on the multifaceted roles of DUBs in PDAC and highlighted the importance of posttranscriptional modifications (PTMs) in cancer progression (Table 1). DUBs affect the proliferation of PDAC cells by regulating stemness, apoptosis, micropinocytosis, and other subphenotypes. Moreover, the invasion and metastasis capabilities of PDAC, even changes in the niches of target organs, are very closely associated with DUBs. The mechanism of chemoresistance and metabolic reprogramming in PDAC is also explained by DUB disorders. The intrinsic character of the immune-suppressive microenvironment in PDAC has resulted in few advances in novel therapeutic strategies for this disease. Emerging studies of DUBs and their impact on PDAC immunity have revealed another potential therapeutic target. Soon, additional studies of DUBs and their effects on the progression of PDAC could lead to the development of more effective methods for the diagnosis and treatment of PDAC in the future.

**Table 1:** The deubiquitinases involved in the progression of pancreatic cancer.

Malignant behavior	DUBs	Family	Involved pathway, target protein	Role	Animal model	References
Proliferation	USP28	USP	USP28–FOXM1-Wnt/β-catenin pathway axis, FOXM1	oncogene	Xenograft tumor in nude mice	[3]
	USP21	USP	USP21–MAPK3 axis, MAPK3	oncogene	Xenograft tumor in nude mice	[11]
	USP21	USP	USP21–TCF7 axis, TCF7	oncogene	Xenograft tumor in nude mice	[14]
	USP34	USP	USP34–p-AKT and p-PKC axis, p-AKT and p-PKC	oncogene	_	[13]
	USP34	USP	USP34–p38/PRR11 axis	oncogene	Xenograft tumor in nude mice	[82]
	USP5	USP	USP5–FOXM1 axis, FOXM1	oncogene	Xenograft tumor in nude mice	[16]
	USP5	USP	USP5–STAT3 pathway axis	oncogene	Xenograft tumor in nude mice	[80]
	USP9X	USP	USP9X–Mcl-1/ITCH axis	tumor suppressor	Xenograft tumor in nude mice	[19]
	USP9X	USP	USP9X–LATS/YAP and TAZ axis, LATS/ YAP and TAZ	tumor suppressor	_	[63]
	USP9X	USP	_	tumor suppressor	Xenograft tumor in nude mice	[65]
	USP9X	USP	USP9X–LATS2 axis, LATS2	tumor suppressor	Xenograft tumor in nude mice	[73]
	USP22	USP	USP22–PTEN–P21 axis, PTEN	tumor suppressor	Xenograft tumor in nude mice	[23]
	USP22	USP	USP22–DYRK1A axis, DYRK1A	oncogene	Xenograft tumor in nude mice	[35]
	USP22	USP	LncRNA CTBP1–AS2–miR-141-3p–USP22 axis	oncogene	_	[57]
	USP33	USP	USP33–TGFBR2–TGFβ signaling pathway axis, TGFBR2	oncogene	Xenograft tumor in nude mice	[24]
	USP33	USP	USP33–CTNNB1 axis, CTNNB1	oncogene	_	[58]
Proliferation	UCHL5	UCHs	UCHL5–ELK3 axis, ELK3	oncogene	Xenograft tumor in nude mice	[25]
	USP4	USP	USP4–TRAF6 axis, TRAF6	oncogene	_	[26]
	USP18	USP	USP18–Notch-1–c-Myc pathway axis, Notch-1	oncogene	Xenograft tumor in nude mice	[28]
	USP10	USP	USP10–SOX21 axis, SOX21	oncogene	Xenograft tumor in nude mice	[30]
	USP10	USP	USP10–PABPC1–CLK2 and eIF4G1 axis, PABPC1	oncogene	Xenograft tumor in nude mice	[72]
	USP10	USP	USP10–BiP–PERK and IRE1α axis	oncogene	_	[86]
	ATXN3	MJD	ATXN3–HDAC6 axis, HDAC6	oncogene	Xenograft tumor in nude mice	[34]
	USP43	USP	USP43–chemokine pathway axis	oncogene	_	[49]
	USP39	USP	miR-133a–USP39–AKT pathway axis	oncogene	Xenograft tumor in nude mice	[56]
	BAP1	UCHs	BAP1–LATS–YAP and TAZ axis, LATS	tumor suppressor	Xenograft tumor in nude mice	[64]
	ATXN3	MJD	ATXN3–YAP1 axis, YAP1	oncogene	_	[91]
Invasion and Metastasis	OTUD1	OTUs	OTUD1–FOXM1 axis, FOXM1	oncogene	Tail vein injection model	[8]
	USP14	USP	USP14–TAZ axis, TAZ	oncogene	Xenograft tumor in nude mice	[9]
	USP9X	USP	USP9X–Snail, Twist, N-cadherin, E-cadherin and vimentin axis	oncogene	_	[18]
	USP10	USP	LINC00460–USP10–p53 axis, p53	oncogene	Xenograft tumor in nude mice	[21]
	USP10	USP	USP10–YAP1–Cyr61 axis, YAP1	oncogene	Tail vein injection model	[36]
	USP10	USP	miR-103–USP10 axis	oncogene	_	[84]
Invasion and metastasis	USP22	USP	USP22–Ezrin/ the focal adhesion kinase (FAK) pathway axis	oncogene	_	[44]
	USP14	USP	USP14–cyclin D1, PCNA and E-cadherin axis	oncogene	_	[45]
	USP5	USP	USP5–WT1–E-cadherin axis, WT1	oncogene	_	[62]
	USP5	USP	USP5–STAT3 pathway axis	oncogene	_	[77]
	CYLD	USP	CYLD–PrP axis, PrP	oncogene	_	[69]
	MINDY	MINDY	MINDY2–ACTN4–PI3K/AKT/mTOR pathway axis, ACTN4	oncogene	Xenograft tumor in nude mice	[79]
	COPS6(CSN6)	JAMMs	COPS6–FOXA1 axis, FOXA1	oncogene	Xenograft tumor in nude mice	[99]
Chemoresistance	USP7	USP	USP7–FBP1–DNMT1 axis, FBP1	oncogene	Xenograft tumor in nude mice	[5]
	USP7	USP	_	oncogene	Xenograft tumor in nude mice	[32]
	DUB3, OTUD1	UBP, OTUs	DUB3–Nrf2 and OTUD1–YAP axis, Nrf2 and YAP	oncogene	_	[6]
	USP8	USP	USP8–Nrf2 axis, Nrf2	oncogene	Xenograft tumor in nude mice	[15]
	USP22	USP	USP22–Wnt/β-catenin pathway axis	oncogene	_	[20]
	USP9X	USP	_	oncogene	Xenograft tumor in nude mice	[27]
	USP9X	USP	USP9X–Mcl-1 axis, Mcl-1	oncogene	Xenograft tumor in nude mice	[55]
	USP9X	USP	miR-212–USP9X axis	oncogene	Xenograft tumor in nude mice	[67]
	USP49	USP	USP49–FKBP51–AKT axis, FKBP51	tumor suppressor	Xenograft tumor in nude mice	[53]
Chemoresistance	USP27X	USP	USP27X–Snail1 axis, Snail1	oncogene	Xenograft tumor in nude mice	[54]
	USP44	USP	USP44–FBP1–MAPK pathway axis, FBP1	tumor suppressor	Xenograft tumor in nude mice	[75]
	OTUB1	OTUs	TFAP2A–OTUB1–SLC7A11 axis, SLC7A11	oncogene	Xenograft tumor in nude mice	[76]
	UCHL3	UCHs	UCHL3–FOXM1 axis, FOXM1	oncogene	Xenograft tumor in nude mice	[78]
	USP10	USP	USP10–N1ICD–Notch1 axis, N1ICD	oncogene	Spontaneous mouse model of pancreatic cancer	[88]
Metabolism	USP25	USP	USP25–HIF-1α axis, HIF-1α	oncogene	Xenograft tumor in nude mice	[2]
	USP10	USP	KIF15–USP10–PGK1 axis, KIF15/PGK1	oncogene	Xenograft tumor in nude mice	[10]
	UCHL3	UCHs	UCHL3–LDHA axis, LDHA	oncogene	_	[22]
Immunity	USP8	USP	USP8–PD-L1 axis, PD-L1	oncogene	Xenograft tumor in nude mice	[4]
	USP22	USP	USP22–SUZ12 and EZH2 (PRC2 complex) axis	oncogene	Xenograft tumor in nude mice	[38]
	USP10	USP	_	oncogene	_	[95]

*USP* ubiquitin-specific proteases, *JAMMs* JAMM/MPN domain-associated metallopeptidases, *UCHs* ubiquitin carboxyl-terminal hydrolase, *MJD* Machado–Joseph domain protease.
